# Effect of Ultra-High Pressure Homogenization (UHPH) and Conventional Thermal Pasteurization on the Volatile Composition of Tiger Nut Beverage

**DOI:** 10.3390/foods12040683

**Published:** 2023-02-04

**Authors:** Idoia Codina-Torrella, Joan Josep Gallardo-Chacón, Bibiana Juan, Buenaventura Guamis, Antonio José Trujillo

**Affiliations:** 1 Centre d’Innovació, Recerca i Transferència en Tecnologia dels Aliments (CIRTTA-UAB), TECNIO, XIA, MALTA-Consolider, Department of Animal and Food Science, Facultat de Veterinària (Edifici V), Universitat Autònoma de Barcelona (UAB), Bellaterra, 08193 Cerdanyola del Vallès, Spain; 2 Department of Agri-Food Engineering and Biotechnology, EEABB, Universitat Politècnica de Catalunya (UPC), Parc Mediterrani de la Tecnologia, Campus del Baix Llobregat (Edifici D4), c/Esteve Terradas, 8, 08860 Castelldefels, Spain

**Keywords:** tiger nut beverage, ultra-high pressure homogenization (UHPH), pasteurization, volatile profile, headspace-solid phase microextraction, gas chromatography-mass spectrometry

## Abstract

Tiger nut beverages are non-alcoholic products that are characterized by their pale color and soft flavor. Conventional heat treatments are widely used in the food industry, although heated products are often damaging to their overall quality. Ultra-high pressure homogenization UHPH) is an emerging technology that extends the shelf-life of foods while maintaining most of their *fresh* characteristics. The present work deals with the comparison of the effect of conventional thermal homogenization-pasteurization (H-P, 18 + 4 MPa at 65 °C, 80 °C for 15 s.) and UHPH (at 200 and 300 MPa, and inlet temperature of 40 °C), on the volatile composition of tiger nut beverage. Headspace-solid phase microextraction (HS-SPME) was used for detecting volatile compounds of beverages, which were then identified by gas chromatography-mass spectrometry (GC-MS). A total of 37 different volatile substances were identified in tiger nut beverages, which were primarily grouped into the aromatic hydrocarbons, alcohols, aldehydes and terpenes chemical families. Stabilizing treatments increased the total amount of volatile compounds (H-P > UHPH > R-P). H-P was the treatment that produced the most changes in the volatile composition of RP, while treatment at 200 MPa had a minor impact. At the end of their storage, these products were also characterized by the same chemical families. This study evidenced the UHPH technology as an alternative processing of tiger nut beverages production that minimally modifies their volatile composition.

## 1. Introduction

Tiger nut beverages are non-alcoholic products obtained from the aqueous extract of tiger nut tubers (*Cyperus esculentus* L.), which are produced and consumed worldwide. One of the most consumed is “horchata de chufa”, a Spanish traditional beverage that is characterized by its pale color and soft flavor [1]. These complex dispersions are characterized by a high percentage of carbohydrates (>50%), fat (~2%), fiber (~1%), and a limited content of protein (~1%) [1,2]. Nowadays, the consumption of vegetal beverages is increasing at the global level, so the beverage industry is focused on improving the safety and shelf-life of these highly perishable products. Conventional thermal pasteurization and ultra-high temperature sterilization (UHT) are the most applied treatments for the microbiological and physicochemical stabilization of marketable tiger nut beverages with an extended shelf-life. However, according to the severity of these treatments, the *fresh* product results in an undesirable loss of its most appreciated sensory characteristics, such as its pale color and almond-like flavor and taste. After heating, different unpleasant flavors appear in these beverages because of the biochemical reactions activated by temperature (i.e., Maillard reaction, caramelization processes, or fat-oxidation reactions, among others), which in some cases, cause significant inconveniences to the consumers. Some authors have characterized commercial thermal-pasteurized and UHT tiger nut beverages and reported that the volatile profiles of these beverages were mainly composed of aldehydes, alcohols, terpenes, and aromatic compounds [3,4,5]. Off-flavors related to some of these components are pungent, oxidized, burnt, or bitter, among others, the reason why the industry tends to mask them through the addition of different flavorings [6] and look for novel stabilizing processes that preserve the overall characteristics of the untreated product, as well.

Ultra-high pressure homogenization (UHPH) is a novel technology that has been demonstrated to allow the microbial and physicochemical stability of different fluid foodstuffs while maintaining the most nutritional and sensory characteristics of the fresh product [7]). Codina-Torrella et al. [8,9] demonstrated the industrial relevance of UHPH to improve the overall quality and shelf-life of tiger nut beverages. These authors also observed that UHPH caused lesser changes in the sensory profile of the raw beverage (color, flavor, taste, etc.) if compared with the conventional thermal-pasteurization processing. To the best of our knowledge, no study exists to determine the effect of UHPH on the volatile profile of tiger nut beverages. The limited existing data about UHPH-treated vegetal beverages reported that, in general, UHPH processing causes fewer changes in the volatile composition of these products than the observed in beverages treated with conventional thermal technologies [10,11]

To complement the studies conducted to date, this study aimed to determine the effect of conventional thermal-pasteurization and UHPH treatments on the volatile profile of tiger nut beverages. This study would allow the industry to have a better understanding of which aromatic compounds are generated during the storage of these beverages, to evaluate which could be the most suitable treatment of pasteurization to preserve the original volatile profile of this product. At the same time, this work has generated more knowledge about the impact of UHPH technology on the volatile profile of vegetal beverages.

## 2. Materials and Methods

### 2.1. Tiger Nut Beverages Production and Processing

Tiger nuts beverages were produced and processed at the Pilot Plant of Universitat Autònoma de Barcelona (SPTA-UAB), as described by Codina-Torrella et al. [7]. The overall experiment was performed in triplicate.

#### 2.1.1. Tiger Nut Beverages Production

The proportion of tubers:water corresponded to 1:8 (*w*:*w*). After pressing and filtering the ground product, 8% of sucrose (*w*/*w*) was mixed with the liquid extract. This mixture was considered the raw product (RP), which general composition (%, *w*/*w*) corresponded to 12.99 ± 0.18 total solids, 10.30 ± 0.60 nitrogen-free materials, 2.01 ± 0.02 fat, 0.54 ± 0.02 protein, and 0.13 ± 0.01 ash. The composition of RP was not affected by the treatment applied. Before the application of all stabilizing treatments, 0.05% of α-amylase enzyme (Bialfa, Biocon Española, S.A., Franqueses del Vallès, Spain) was added to the RP (holding time of 10 min, at room temperature), to hydrolyze the starch granules. Qualitative determination of starch (Total Starch Assay Procedure kit, Amyloglucosidase/α-amylase method, K-TSTA 404-2009, Megazyme International Ireland Ltd., Wicklow, Ireland) demonstrated that this component was hydrolyzed.

#### 2.1.2. Beverage Treatments: UHPH, Homogenization-Pasteurization

Two different pasteurizing UHPH treatments were performed by using an ultra-high pressure homogenizer, at a flow rate of 120 L/h (Model: DRG No. FPG11300:400 Hygienic Homogenizer, Stansted Fluid Power Ltd., Harlow, UK) at two different pressures, 200 and 300 MPa, and the same inlet temperature (Ti) of 40 °C. The temperature of UHPH-treated beverages increased by 24.2 °C between pressures ranging from 200 to 300 MPa [8]. Temperature after the UHPH valve corresponded to 92.1 ± 1.7 and 116.3 ± 4.3 °C for the 200 and 300 MPa treatments, respectively, and the residence time of the product at these temperatures was estimated to be <0.7 s. The outlet temperature of products corresponded to 15.3 ± 1.1 and 17.1 ± 1.6 °C in 200 and 300 MPa treatments, respectively.

Conventional treatment of Homogenization-Pasteurization (H-P) was also applied to the RP using an indirect heat system composed of a double-stage homogenizer positioned upstream (Model X68, Soavi B. and Figli, S.P.A., Parma, Italy) and a multitube tubular heat exchanger at a flow rate of 1000 L/h (laminar flow) (6500/010, GEA Finnah GmbH, Ahaus, Germany). Beverages were homogenized at pressures of 18 MPa (first stage-valve) and 4 MPa (second stage-valve) at 65 °C, and subsequently pasteurized at 80 °C for a holding time of 15 s. Samples (RP, H-P, 200 MPa, and 300 MPa) were collected in sterile glass bottles of 1 L of capacity with twist-off caps (Apiglass Envases y Material Apícola, S.L., Barcelona, Spain) inside a laminar flow cabin (Mini-V cabin, Telstar Technologies, S.L., Terrassa, Spain) and were stored at refrigeration temperature (4 °C) until their analyses

Microbiological shelf-life of stored beverages corresponded to 3, 5, 30 and 57 days for the RP, H-P and UHPH processed beverages at 200 and 300 MPa, respectively, according to Codina-Torrella et al. [9].

### 2.2. Procedure of HP-SPME and GC-MS

Tiger nut’s beverage samples were evaluated after production and during their previously established shelf-life. Volatile compounds were analyzed following the method optimized by Klein et al. [4], with some modifications. All analyses were performed in triplicate.

#### 2.2.1. HP-SPME Extractions

An aliquot of 2 mL of each beverage was placed in different vials with 3 μL of an internal standard (4-methyl-2-pentanol in methanol, 5 ppm). Vials were incubated for 10 min at 40 °C for their stabilization, at continuous homogenization (stirring with a magnetic stirrer at 700 rpm). After sample stabilization, SPME fiber of 85 μm (DVB/CAR/PDMS, Supelco, Bellefonte, PA, USA) was exposed to the vial headspace, for 30 min at 40 °C, in which volatile compounds were adsorbed.

#### 2.2.2. GC-MS Analysis

Adsorbed volatiles were desorbed in the gas chromatograph (GC) injector port, in splitless mode, at 250 °C for 3 min. The split valve was opened, and the fiber was kept in the injector for 15 min for a cleaning step. The headspace of the volatile compounds was analyzed using an automated GC (model: 6890 Series II, Agilent, Santa Clara, CA, USA). The analysis was carried out on a 60 m × 0.25 mm internal diameter capillary column, with a film thickness of 0.25 μm (TRB-Wax, Agilent technologies). The mass spectrometry (MS) selective detector (model: 5972 Agilent, Santa Clara, CA, USA) was used in electron impact ionization mode with a mass range of 30–250 m/z. Before each analysis, the fiber was preconditioned for 1 h at 250 °C. The temperature was programmed in two stages. The initial temperature was kept at 40 °C for 5 min, and then, it increased at the rate of 10 °C/min to the temperature of 250 °C and held for 10 min.

Tentative identification of volatile compounds was achieved by comparing their mass spectra with those of the mass spectra libraries Willey 1n.l and NIST 0.5 (National Institute of Standards and Technology). Relative retention times of detected compounds were also determined by injecting 1 μL of alkane standard solutions (Alkane standard solution C8–C20 from Sigma–Aldrich and Connecticut ETPH calibration mixture C9–C36, with purity greater than 95%) in triplicate with a split ratio of 1:200. Signals were processed using Agilent MSD Productivity ChemStation Enhanced Data Analysis software (Agilent, Santa Clara, CA, USA). Confirmation of the identification of hexanal, pentanal, 1-octen-3-one, 2,3-pentanodione, 1-hexanol, 1-pentanol, 1-octen-3-ol and 2-penthyl furan (Sigma-Aldrich, St. Louis, MO, USA) was performed by comparing GC retention times and mass spectra of individual components with those authentic reference compounds injected under the same conditions. The results of volatile compounds were expressed as microgram (µg) equivalents of 4-methyl-2-pentanol internal standard per milliliter (mL) of tiger nuts’ milk beverage. The limit of quantification (LOQ) of metabolites was also determined by measuring the average noise and the standard deviation values of 10 blanks (calibration matrix). Average noise plus 10 times standard deviation was used for LOQ. Relative standard deviation (RSD) was calculated for five replicate measurements of each compound solution. Only compounds with RSD <10% were finally considered.

### 2.3. Statistical Analysis

One-way analysis of variance (ANOVA) was performed on volatile compounds, by using the GLM procedure of Statgraphics (Statgraphics Inc., Chicago, IL, USA). Tukey test was used for the data comparison and significant differences were determined at the 5% level of probability. Principal component analysis (PCA) was performed to reduce the data in two dimensions and identify patterns of variation in the results. R software (R software, Auckland, New Zealand) was used for this purpose. In this paper, data showed corresponds to the mean ± standard error.

## 3. Results and Discussion

### 3.1. Effect of Treatments on the Volatile Composition of Beverages

Analysis of GC-MS of all beverages revealed about 37 different volatile substances in tiger nut beverages (Table 1). RP was characterized by aromatic hydrocarbons and alcohols, which represented ~52.3 and ~36.2% of the total, respectively, followed in importance by aldehydes (~6.4% of the total) and terpenes (~3.7% of the total). The main volatile compounds found in this sample were toluene, ethanol, 1-octanol, 1-nonanol, nonanal, and limonene. The significant presence of aromatic hydrocarbons in tiger nut beverages could be attributed to different origins, such as the breakdown of plant carotenoids, the uptake by plants of chemical substances from the environment, or due to the traditional practice of burning the aerial part of the plant before harvesting of tubers [12,13,14]. It is also reported in the literature that alcohols are one of the most representative aromatic groups of raw tiger nuts (~68%), followed by aldehydes (~6.4%), pirazines (~5.6 %), and terpenes (~4.2%) [15].

As shown in Table 1, the application of stabilizing treatments to the RP increased the total amount of volatile compounds in this sample, according to H-P > UHPH > R-P (*p* < 0.05). H-P beverage was characterized by aromatic hydrocarbons (~48.7%) and alcohols (~37.5%), followed by aldehydes (~6%) and another minor groups of components (~7.3%) represented by terpenes, furans, ketones, esters, phenolic compounds, and acids. Some of these compounds were probably formed during the sample exposure to the temperature, which enhances lipid oxidation and browning reactions [16]. UHPH-treated beverages at 200 and 300 MPa were also characterized by aromatic hydrocarbons (50.3 and 57.5%, respectively) and alcohols (41.7 and 32.2%, respectively), but in this case, terpenes took the third place in importance (5.9 and 5.7%, respectively). Comparing both UHPH-treated beverages, higher amounts of ketones and aldehydes were observed in the 300 MPa sample, which was mainly attributed to the synergic effect of the higher temperature reached after the high-pressure valve (~92 and ~116 °C, respectively in 200 and 300 MPa treatments) and the homogenization pressure onto the food matrix’s components.

#### 3.1.1. Main Groups of Volatile Patterns: Aromatic Hydrocarbons, Alcohols and Aldehydes

Aromatic hydrocarbons were the most detected compounds of beverages, according to H-P ≥ 200 MPa = 300 MPa ≥ RP (Table 1). In all samples, toluene was the most representative compound, followed by the isomers of xylene, ethylbenzene and styrene. On the contrary, m-cymene was only identified in UHPH-treated beverages. These components, which have been associated with earthy and musty off-flavors, are described as an important contaminant group of processed food, and their formation is related to heating and several processing techniques [17,18].

Alcohols and aldehydes were the second and third most important chemical groups, respectively, of all tiger nut beverages. Their presence was significantly affected by the treatment applied (*p* < 0.05), according to H-P > 200 MPa = 300 MPa > RP. These compounds are broadly used as quality markers of oils because their formation is associated with fatty acid oxidation, among others [19]. The most representative alcohols of tiger nut beverages were 1-nonanol, 1-octanol and ethanol (Table 1). UHPH samples also presented a high content of 1-hexanol, which might originate from the linoleic acid degradation, and, as reported in the literature, their presence is associated with bitter and floral aromas [20]. Homogenized sample at 200 MPa was characterized by the highest content of 1-octen-3-ol, which is associated with mushroom aromas [16].

Concerning the aldehydes, three different compounds were detected in the beverages evaluated in this study, which corresponded to hexanal, octanal and nonanal. Cantalejo (1997) [15] had previously reported that total aldehydes represented the ~6.4 % of total volatile compounds in tiger nuts, of which benzaldehyde was the most abundant (5%), followed by hexanal, nonanal, and octanal. Nonanal was the most abundant compound of RP, H-P, and 200 MPa beverages (H-P > RP > 200 MPa), with values around 89, 89, and 97.8% of total aldehydes, respectively. On the contrary, the most representative aldehyde in the 300 MPa sample was hexanal (~48% of the total), followed by octanal (~31% of the total). As observed, the H-P treatment caused the most significant increase in the total amount of aldehydes, which could be explained by the highest lipid oxidation reactivity due to heat processing (Table 1). The 200 MPa-treated beverages presented the lowest values, which suggested that this treatment could improve the oxidative stability of the RP. The presence of these compounds is commonly related to grassy, green, and beany flavors [21,22,23]. Although UHPH-treated products might be expected to be oxidized faster (due to the increase in the number of fat particles and the subsequent increase of total fat surface exposed to oxidation [24], new interactions created during the UHPH process between denatured proteins and the other components of the dispersion might result in a significant protective effect against fat droplet’s oxidation [25]. Differences observed between both UHPH-treated samples (200 and 300 MPa) were probably attributed to the higher temperature reached during the treatment [8].

Technological treatments increased substantially (*p* < 0.05) the presence of total terpenes in samples, not showing significant differences between treated beverages. Terpenes are ubiquitous compounds of vegetables, which are produced during their metabolism to fight pests and other diseases [26]. Limonene was the most relevant terpene in all samples, and which content increased significantly (*p* < 0.05) after processing. In line with this, other authors also detected limonene as the predominant terpene in tiger nut beverages [4,6]. Badui (2006) [27] suggested that heat treatments could affect the compounds that keep emulsified limonene, which therefore let it release. The increase of terpenes in UHPH beverages could be related to the effect of pressure on limonene liberation. To a lesser extent, other terpenes were detected in these beverages, such as β-pinene in both UHPH-treated beverages, α-pinene and γ-terpinene in RP and UHPH beverages, and 1-α-terpineol in H-P and UHPH samples. The presence of these compounds is associated with pine, citric, or musty aromas [27,28].

#### 3.1.2. Secondary Compounds of Beverages: Ketones, Acids, Esters, Phenolic Compounds, and Furans

Five different ketones were detected in treated tiger nut beverages (Table 1). Their highest presence was detected in the 300 MPa sample, whereas no differences were observed between H-P and 200 MPa samples (*p* > 0.05). Contents of 6-methyl-5-hepten-2-one and 1-phenylethanone were not affected by the treatment (*p* > 0.05), while 2-nonanone and 1-etanone contents increased after 300 MPa and H-P processes. Ketones are described to be derived from Maillard, Strecker, and lipid oxidative reactions [29,30], and their presence had been described in tiger nuts by-products after roasting [15,31]. In this study, ketones were not detected in the RP, probably due to their under-representation in raw tubers (~0.68 of total volatile compounds). Klein et al. (2014) [4] also identified eight different ketones in conventional pasteurized and sterilized tiger nut beverages, the amount of which also increased according to the severity of the heat treatment. In the literature, these volatile compounds had been related to kindly aromas (caramel, sweet, fruit-like, or buttery) but also with mushroom or green-beany unpleasant flavors [16,30].

Acids, esters, phenolic compounds, and furans corresponded to the minor groups of volatile compounds in all samples, and their prevalence increased (*p* < 0.05) after H-P, if compared with beverages treated by UHPH (Table 1). Three different acids (hexanoic, benzoic, and butanoic) were identified in the RP, of which benzoic and butanoic increased significantly (*p* < 0.05) after H-P treatment. Hexanoic acid was not detected in the H-P beverage. On the contrary, UHPH treatment at 200 MPa did not cause significant differences (*p* < 0.05) in the percentage of the total acids, in comparison to the RP (only butanoic acid was detected in this sample), and no acids were detected in the beverage homogenized at 300 MPa. Among others, the contribution of acids in food aroma has been related to cheese and acidic odors [16]. Concerning the esters, ethylcaprylate was only detected in the sample treated by heat pasteurization. It had been previously reported the low prevalence of esters in raw tiger nuts (1.45% of the total), of which ethylcaprylate represented ~0.1% of the total [15]. In the current study, only one furan (2-pentyl furan) was detected in RP and H-P samples, according to RP < H-P (*p* < 0.05) (Table 2). Lower amounts of phenolic compounds were identified in all treated beverages. H-P beverages showed the highest content, probably due to the heat degradation of phenolic acids from tiger nuts (such as tannins). 4-vinil-2-metoxyphenol was identified in all beverages, according to RP > UHPH ≥ RP. On the contrary, phenol-2-metoxy and phenol were only detected in H-P and UHPH samples, respectively. According to the literature, the presence of phenolic acids in food is related to green and harsh grassy odors [32,33]. Furans are widely associated with heating, which origin is reported to be related to Maillard reactions and the oxidation processes of unsaturated fatty acids [23]. Cantalejo (1997) [15] isolated different furans in raw tiger nuts, which increased in number and concentration after the tuber’s roasting, and Klein et al. (2014) [4] also identified four types of furans in different commercial UHT Tiger nut beverages. No furans were detected in both UHPH-treated beverages, probably due to the lesser effect of temperature during this process, in comparison to the H-P. Among others, these compounds had been related to buttery, almond-like, sweet, and green bean-like odors [34,35].

### 3.2. Principal Component Analysis (PCA)

**Figure 1:**
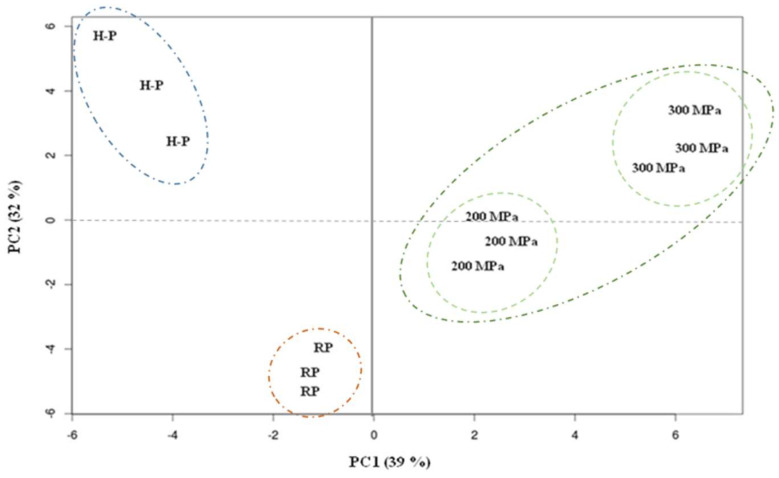
Loadings plot after principal component analysis of the individuals in the plane defined by the two first principal components (PC1 and PC2). PC1 (39%): First Principal Component, which explains 39% of total variability; PC2 (32%): Second Principal Component, which explains 32% of total variability; RP: raw product; H-P: homogenization-pasteurization at 18 + 4 MPa (pressure of first stage valve + second stage valve) and 65, and 80 °C for 15 s; 200 MPa: ultra-high pressure homogenization at 200 MPa and Ti of 40 °C; 300 MPa: ultra-high pressure homogenization at 300 MPa and Ti of 40 °C.

Figure 1 shows the distribution of samples in the principal components 1 (PC1) and 2 (PC2). This clustering method reduces the dimensionality of multivariate data and preserves the variance therein [36]. In this study, PC1 and PC2 explained 71% of the global variability of the dataset (Table 2). As observed in Figure 1, samples were perfectly distributed along these two axes in three main groups differentiated by the stabilizing process.

PC1 and PC2 scores separate the different samples of beverages (Table 2). As shown in Figure 1, UHPH samples could be grouped in the same group, although these two samples also presented some differences. PC1 could be entirely related to the treatment. The highest negative scores (which represent strong influence) in PC1 are detected in some aromatic hydrocarbons, two alcohols (ethanol and 2-octanol), 4-viny-2-metoxyphenol, 2-pentil-furan and nonanal, which are related to heat-damage effects and separate treated beverages through the axis. In this component, large positive loadings in two ketones (3-octanone and 2-nonanone) and 1-octen-3-ol were principally influenced by 300 and 200 MPa treatments, respectively, while high loadings for hexanal and two terpenes (α and β-pinene) were linked with the effect of UHPH. Concerning PC2, large positive scores in aromatic hydrocarbons, aldehydes, two alcohols (1-hexanol, 1-nonanol), 1-phenylethanone, and limonene were identified as markers of treated samples (H-P and UHPH). Large negative scores of γ-terpiene and hexanoic acid were related to the RP, which contributed to differentiating this untreated beverage from the others.

According to their volatile profile, 200 MPa and RP beverages would be the most similar products, followed by the 300 MPa and the H-P samples. Codina-Torrella et al. [8] reported previously that 200 MPa treatment caused lesser changes in the physicochemical characteristics (color, viscosity, oxidation reactions) of RP, compared with changes observed in the raw base after 300 MPa and H-P treatments.

### 3.3. Changes in the Volatile Profile of Beverages during Their Storage

**Figure 2:**
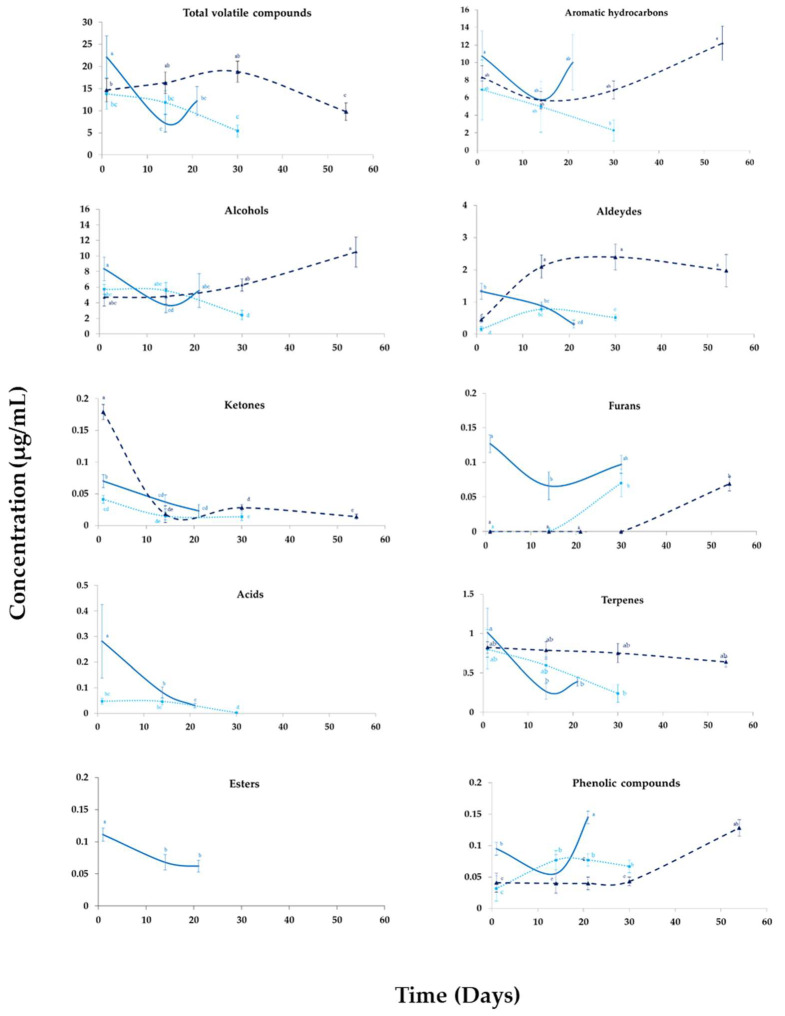
Evolution of volatile compounds in tiger nut beverages during their storage at 4 °C. ^a–d^ For each group of family compounds, values with different letters differed significantly (*p* < 0.05). H-P: homogenization-pasteurization at 18 + 4 MPa (pressure of first stage valve + second stage valve) at 65, and 80 °C for 15 s (-); 200 MPa: ultra-high pressure homogenization at 200 MPa and Ti = 40 °C (…); 300 MPa: ultra-high pressure homogenization at 300 MPa and Ti = 40 °C (----); Concentration: µg equivalents of 4-methyl-2-pentanol internal standard per mL of tiger nuts’ milk beverage.

Figure 2 shows the evolution of each family group of volatile compounds of stored beverages. The volatile profile of RP was not evaluated since its shelf-life only corresponded to 3 days. During their storage, the total amount of volatile compounds showed a significant decrease (*p* < 0.05) in all pasteurized beverages. Secondary products generated from lipid oxidation reactions, non-enzymatic browning reactions (Maillard and caramelization), and the microbiological evolution of each sample probably explained the differences observed between these beverages and their corresponding homologs after production. At the end of the storage time, the aromatic profiles of tiger nut beverages were represented by the aromatic hydrocarbons, alcohols, aldehydes, and terpenes families. In contrast to H-P and 200 MPa samples, the alcohols increased in 300 MPa beverage at the end of the storage time. These changes were probably attributed to the reaction of aldehydes and ketones derived from lipid oxidative reactions that occurred in the sample over time. Aldehydes decreased in importance in H-P beverages (Figure 2), while in both UHPH beverages, their content increased significantly (*p* < 0.05). Homogenized beverages at 300 MPa showed the highest content of aldehydes. Our previous research [9] suggested faster oxidation reactions in 300 MPa samples, probably due to the increase of total fat surface by the increase in the number of oil droplets after the homogenization process. Terpenes only increased in the 300 MPa sample (Figure 2). Concerning the minority groups of volatile compounds, total furans and phenolic compounds increased in all beverages, although their content in the H-P sample was significantly higher than in their homologs. On the contrary, ketones, acids, and esters decreased in all beverages over time (Figure 2), which demonstrated that these aromatic family groups only were involucrate in the sensory profile of these beverages at the beginning of their shelf-lives.

## 4. Conclusions

In this study, a total of 37 different volatile substances were identified in tiger nut beverages. Raw product (RP) was characterized by aromatic hydrocarbons and alcohols, which represented ~52.3 and ~36.2% of the total, respectively, followed in importance by aldehydes (~6.4% of the total) and terpenes (~3.7% of the total). The application of stabilizing treatments of homogenization-pasteurization (H-P and UHPH) increased the total amount of volatile compounds of samples, in comparison to the RP, according to H-P > UHPH > R-P. H-P treatment induced the most important changes in the raw base, while treatment at 200 MPa had a minor impact. H-P-treated beverage was characterized by aromatic hydrocarbons (~48.7%) and alcohols (~37.5%), followed by aldehydes (~6%). On the contrary, UHPH-treated beverages at 200 and 300 MPa were characterized by aromatic hydrocarbons (50.3 and 57.5%, respectively) and alcohols (41.7 and 32.2%, respectively), but in this case, terpenes took the third place in importance (5.9 and 5.7%, respectively). Differences observed in both UHPH samples were probably attributed to the synergic effect of pressure and temperature. During the storage time, the total amount of volatile compounds decreased in all beverages. The volatile profile of these products was also characterized by the high content of alcohols, aldehydes, aromatic hydrocarbons, and terpenes. Results obtained in this study evidenced that H-P treatment has a greater impact on the chemical volatile profile of tiger nut beverages if compared with the UHPH process. According to this, UHPH technology could be proposed as an alternative processing of pasteurization in tiger nut beverages production to obtain *fresh like* products.

## Figures and Tables

**Table 1:** Concentration of volatile compounds (µg of 4 methyl-2-pentanol/mL) in tiger nut beverages, grouped by their chemical family.

Family Group	Compound	ID ^1^	KI ^2^	KI lit. ^3^	Treatment ^4^			
					RP	H-P	200 MPa	300 MPa
Aromatic Hydrocarbons	Toluene	MS, IR, S	1053.0	1042	3.419 ± 0.961 ^b^	9.835 ± 1.567 ^ab^	6.182 ± 1.949 ^ab^	7.424 ± 0.738 ^ab^
	Ethylbenzene	MS, IR, S	1132.2	1124	0.028 ± 0.002 ^b^	0.048 ± 0.009 ^ab^	0.086 ± 0.018 ^a^	0.089 ± 0.007 ^a^
	p-xylene	MS, IR, S	1141.4	1150	0.013 ± 0.001 ^c^	0.199 ± 0.050 ^a^	0.036 ± 0.003 ^b^	0.068 ± 0.003 ^b^
	m-xylene	MS, IR	1146.3	1150	0.071 ± 0.006 ^b^	0.216 ± 0.039 ^a^	0.247 ± 0.048 ^a^	0.289 ± 0.018 ^a^
	o-xylene	MS, IR	1191.9	1182	0.036 ± 0.002 ^c^	0.167 ± 0.030 ^b^	0.114 ± 0.020 ^b^	0.213 ± 0.017 ^a^
	Styrene	MS, IR	1256.5	1273	0.024 ± 0.012 ^c^	0.061 ± 0.011 ^ab^	0.076 ± 0.024 ^a^	0.030 ± 0.003 ^b^
	m-cymene	MS, IR, S	1288.4	1267	<LOQ	<LOQ	0.096 ± 0.025 ^a^	0.085 ± 0.010 ^a^
	1,2,4-trimethylbenzene	MS, IR	1295.3	1293	0.002 ± 0.000 ^c^	0.183 ± 0.032 ^a^	<LOQ	0.092 ± 0.005 ^b^
	Naftalene	MS, IR	1795.7	1825	<LOQ	0.022 ± 0.004 ^ab^	0.029 ± 0.003 ^a^	0.018 ± 0.003 ^b^
	Total				3.593 ± 1.961 ^b^	10.731 ± 2.805 ^a^	6.866 ± 3.424 ^ab^	8.308 ± 1.354 ^ab^
Alcohols	Ethanol	MS, RI	941.4	936	0.523 ± 0.181 ^c^	1.924 ± 0.572 ^a^	0.823 ± 0.243 ^b^	0.490 ± 0.127 ^c^
	1-hexanol	MS, RI, S	1357.6	1355	0.105 ± 0.022 ^c^	0.192 ± 0.013 ^b^	0.355 ± 0.027 ^a^	0.295 ± 0.031 ^a^
	2-octanol	MS	1417.8	1421	<LOQ	0.034 ± 0.005 ^a^	<LOQ	<LOQ
	1-octen-3-ol	MS, RI, S	1451.9	1451	<LOQ	<LOQ	0.272 ± 0.117 ^a^	<LOQ
	1-heptanol	MS	1454.8	1455	0.096 ± 0.010 ^c^	0.273 ± 0.023 ^b^	<LOQ	1.295 ± 0.184 ^a^
	1-octanol	MS, RI. S	1549.2	1565	0.421 ± 0.025 ^b^	1.285 ± 0.306 ^a^	1.167 ± 0.081 ^a^	0.013 ± 0.001 ^c^
	1-nonanol	MS, RI	1659.2	1661	1.339 ± 0.020 ^c^	4.560 ± 0.652 ^a^	3.067 ± 0.151 ^b^	2.570 ± 0.314 ^bc^
	Total				2.484 ± 0.109 ^b^	8.268 ± 1.522 ^a^	5.684 ± 0.632 ^a^	4.664 ± 1.111 ^ab^
Phenolic Compounds	Phenol-2-metoxy	MS, RI	1889.8	1883	<LOQ	0.007 ± 0.002 ^a^	<LOQ	<LOQ
	Phenol	MS, RI, S	2024.8	2209	<LOQ	<LOQ	0.018 ± 0.002 ^b^	0.037 ± 0.015 ^a^
	4-vinil-2-metoxyphenol	MS, RI	2227.7	2223	0.034 ± 0.003 ^b^	0.088 ± 0.022 ^a^	0.014 ± 0.001 ^c^	0.004 ± 0.001 ^d^
	Total				0.034 ± 0.003 ^b^	0.095 ± 0.005 ^a^	0.032 ± 0.002 ^b^	0.041 ± 0.007 ^b^
Aldehydes	Hexanal	MS, RI, S	1089.4	1098.0	0.011 ± 0.001 ^c^	0.028 ± 0.003 ^bc^	0.045 ± 0.012 ^b^	0.212 ± 0.0253 ^a^
	Octanal	MS, RI, S	1296.9	1299	0.037 ± 0.004 ^bc^	<LOQ	0.047 ± 0.007 ^b^	0.139 ± 0.009 ^a^
	Nonanal	MS, RI, S	1406.2	1394	0.389 ± 0.400 ^b^	1.303 ± 0.192 ^a^	0.071 ± 0.022 ^c^	0.093 ± 0.015 ^c^
	Total				0.437 ± 0.079 ^b^	1.331 ± 0.341 ^a^	0.163 ± 0.072 ^c^	0.444 ± 0.082 ^b^
Terpenes	α- pinene	MS, RI	1028.7	1032	0.030 ± 0.003 ^b^	<LOQ	0.040 ± 0.008 ^a^	0.037 ± 0.005 ^a^
	β- pinene	MS, RI	1104	1113	<LOQ	<LOQ	0.009 ± 0.002 ^a^	0.006 ± 0.001 ^a^
	Limonene	MS, RI, S	1195.9	1203	0.166 ± 0.011 ^b^	0.975 ± 0.185 ^a^	0.722 ± 0.136 ^a^	0.750 ± 0.022 ^a^
	γ-terpiene	MS, RI	1246.1	1178	0.059 ± 0.016 ^a^	<LOQ	0.014 ± 0.002 ^b^	0.018 ± 0.003 ^b^
	l-α-terpineol	MS, RI	1708.8	1719	<LOQ	0.036 ± 0.007 ^a^	0.016 ± 0.004 ^b^	0.012 ± 0.001 ^b^
	Total				0.255 ± 0.018 ^c^	1.011 ± 0.311 ^a^	0.801 ± 0.251 ^ab^	0.823 ± 0.073 ^ab^
Ketones	3-octanone	MS, RI	1266.1	1265.5	<LOQ	<LOQ	<LOQ	0.005 ± 0.000 ^a^
	6-methyl-5-hepten-2-one	MS, RI, S	1351.0	1342	<LOQ	0.017 ± 0.005 ^a^	0.022 ± 0.001 ^a^	0.027 ± 0.001 ^a^
	2-nonanone	MS, RI, S	1399.3	1436	<LOQ	0.012 ± 0.001 ^b^	<LOQ	0.123 ± 0.007 ^a^
	1-phenylethanone	MS, RI	1684.7	1650	<LOQ	0.025 ± 0.002 ^a^	0.019 ± 0.003 ^a^	0.024 ± 0.002 ^a^
	1-ethanone	MS	1903.2		<LOQ	0.016 ± 0.004 ^a^	<LOQ	<LOQ
	Total				<LOQ	0.070 ± 0.021 ^b^	0.041 ± 0.015 ^b^	0.179 ± 0.012 ^a^
Acids	Hexanoic	MS, RI	1236.3	1244	0.003 ± 0.000 ^a^	<LOQ	<LOQ	<LOQ
	Butanoic	MS, RI	1681.6	1638	0.014 ± 0.005 ^b^	0.246 ± 0.059 ^a^	0.048 ± 0.020 ^b^	<LOQ
	Benzoic	MS	1821.1		0.015 ± 0.002 ^b^	0.036 ± 0.006 ^a^	<LOQ	<LOQ
	Total				0.032 ± 0.005 ^b^	0.282 ± 0.143 ^a^	0.048 ± 0.011 ^b^	<LOQ
Esters	Etilcaprilate	MS, RI	1444.3	1444	<LOQ	0.111 ± 0.010 ^a^	<LOQ	<LOQ
Furans	2-pentil furan	MS, RI, S	1234.2	1244	0.028 ± 0.002 ^b^	0.127 ± 0.013 ^a^	<LOQ	<LOQ
TOTAL					6.86 ± 0.25 ^c^	22.03 ± 1.52 ^a^	13.63 ± 3.73 ^b^	14.46 ± 2.30 ^b^

^a–d^ Values in the same row with different letters differed significantly (*p* < 0.05); ^1^ ID: Identification, MS = mass spectra, RI = retention index as reported in Pherobase and Flavomet databases, S = positively identified by comparison with authentic standards; ^2^ KI: Kovats retention index calculated; ^3^ KI lit.: Kovats retention index reported in the literature; ^4^ Treatment: RP = raw product, H-P = homogenization-pasteurization at 18 + 4 MPa (pressure of first stage valve + second stage valve) at 65, and 80 °C for 15 s, 200 MPa = ultra-high pressure homogenization at <200 MPa and Ti of 40 °C, 300 MPa = ultra-high pressure homogenized product, at 300 MPa and Ti of 40 °C; LOQ: Limit of quantification.

**Table 2:** Percentage variance and loading accounted by the first two principal components (PC1 and PC2) of tiger nut beverages’ volatile profile.

Name	Principal Components ^1^	
	PC1	PC2
Aromatic Hydrocarbons		
Toluene	0.045	0.248
Ethylbenzene	0.189	0.148
p-xylene	−0.062	0.236
m-xylene	0.178	0.182
o-xylene	0.152	0.204
Styrene	−0.081	0.070
m-cymene	−0.165	0.135
1,2,4-trimethylbenzene	0.130	0.071
Naftalene	0.065	0.196
Aldehydes		
Hexanal	0.205	0.122
Octanal	0.123	0.196
Nonanal	−0.207	0.117
Alcohols		
Ethanol	−0.169	0.127
1-hexanol	0.122	0.216
2-octanol	−0.184	0.100
1-octen-3-ol	0.184	0.180
1-heptanol	0.052	−0.036
1-octanol	−0.165	0.085
1-nonanol	−0.016	0.271
Phenolic Compounds		
Phenol-2-metoxy	−0.164	0.154
Phenol	0.196	0.063
4-vinil-2-metoxyphenol	−0.221	0.106
Ketones		
3-octanone	0.205	0.127
6-methyl-5-hepten-2-one	−0.108	0.070
2-nonanone	0.221	0.096
1-phenylethanone	0.014	0.244
1-etanone	−0.157	0.162
Terpenes		
α- pinene	0.232	−0.056
β- pinene	0.208	0.067
Limonene	0.080	0.243
γ-terpiene	0.046	−0.215
l-α–terpineol	−0.023	0.219
Acids		
Hexanoic	−0.061	−0.209
Benzoic	−0.219	0.076
Butanoic	−0.161	0.171
Furans		
2-pentil furan	−0.206	0.121
Esters		
Etilcaprilate	−0.148	0.178
Variance explained (%)	39	32

^1^ PC1: First Principal Component, PC2: Second Principal Component.

## Data Availability

Data is contained within the article.
